# Supplementary material for: COVID-19 vaccine uptake, confidence and hesitancy in rural KwaZulu-Natal, South Africa between April 2021 and April 2022: A continuous cross-sectional surveillance study
Source: PLOS Glob Public Health. 2023 Jun 27;3(6):e0002033. doi: 10.1371/journal.pgph.0002033 (PMC10298801; doi:10.1371/journal.pgph.0002033)
Supplement: S1 Table — (DOCX) [file pgph.0002033.s002.docx]

**Supplementary Material**

Title: COVID-19 vaccine uptake, confidence and hesitancy in rural KwaZulu-Natal, South Africa between April 2021 and April 2022: a continuous cross-sectional surveillance study

**Authors**: Rachael Piltch-Loeb, Lusanda Mazibuko, Eva Stanton, Thobeka Mngomezulu, Dickman Gareta, Siyabonga Nxumalo, John D. Kraemer, Kobus Herbst, Mark J. Siedner, Guy Harling

**Supplementary Table 1. Independent variable definitions**

|  | **Questions as asked** | **Edits for analysis** |
| --- | --- | --- |
| ***Demographics*** |  |  |
| Gender | Male; female |  |
| Age | Continuous age in years | Four categories based on timing of vaccine eligibility: 18-3, 35-49, 50-59. 60+ |
| ***Contextual*** |  |  |
| COVID information sources | Single question with 12 response options, allowing multiple responses: “Where do you get information about COVID-19 that you trust?” | Four binary variables (yes if affirm any in category): 1) Traditional (newspapers, radio, TV, government websites); 2) Personal network (social media, email/WhatsApp, friends/family); 3) Healthcare (clinics/healthcare workers); 4) Community (traditional healers, school, church, community leaders) |
| Mistrust in government | Three questions asking about agreement with statements, responses on 5-point Likert scale: “The government cannot be trusted to tell the truth about COVID-19” “Government is not giving enough clear information about COVID-19” “Information about coronavirus is being withheld from the public” Response options: Strongly agree; Agree; Neither agree nor disagree; Disagree; Strongly disagree | Sum of responses (max 12), z-score standardized across all respondents |
| Educational attainment | Highest grade attainment | Categorized by educational level: none; primary; some secondary; completed secondary; any tertiary |
| Urbanicity | Household defined by geographic location as: rural, peri-urban, urban |  |
|  |  |  |
| ***Group/community influence*** |  |  |
| Household economic change | Single question with response options on a five-point Likert scale: “Overall, how do you feel your household’s current financial situation compares to before COVID-19: Much worse off; A little worse off; About the same; A little better off; Much better off?” |  |
| Community wellbeing | Single question with three categorical response options: “Since March 2020, do you feel things in general in your community have: got better, stayed the same, got worse?” |  |
| Household vulnerability | Continuous age in years for each household member | Binary: any household member aged over 60 |
| COVID stereotype stigma | Six questions asking about agreement with statements, responses on 4-point Likert scale: 1) “People with coronavirus did something wrong and deserve to be punished”; 2) “People with coronavirus are irresponsible”; 3) “People with coronavirus bring shame on their families”; 4) “People with coronavirus are dirty”; 5) “People with coronavirus are cursed”; 6) “If someone you knew had coronavirus and recovered, you would be afraid to visit them. Response options: Strongly disagree; Disagree; Agree; Strongly agree | Sum of responses (max 18), z-score standardized across all respondents |
| COVID anticipated stigma | Five statements about agreement with statements, responses on 5-point Likert scale: “Think about how other people would treat you if you were to get coronavirus. How likely is it that people would treat you in the following ways: 1) A friend or family member would be angry with me; 2) A friend or family member will blame me for getting sick; 3) A friend or family member will think it was my fault that I got sick with coronavirus; 4) It would be hard to tell other people; 5) I would feel ashamed”. Response options: Strongly disagree; Disagree; Neither agree nor disagree; Agree; Strongly agree | Sum of responses (max 20), z-score standardized across all respondents |
| ***Cues to action*** |  |  |
| Concern if acquired COVID | Single question with 4 categorical response options: “How concerned are you about your exposure to COVID-19: Not at all; Slightly concerned; Moderately concerned; Very concerned? |  |
| Knowledge of others with COVID | Single question with 3 categorical answers: “Do you know anyone who has been diagnosed as having COVID-19: Yes; No; Don’t know?” | Binary: yes vs any other response |
| Household vaccination behaviour | Self- and proxy-reported vaccine uptake (any, ever) for all resident household members | Binary: any other household member reported vaccinated up and including the date of interview |
| Mental health | Four questions comprising PHQ-4 (Kroenke et al, 2009; <https://doi.org/10.1016/S0033-3182(09)70864-3>):  “Over the last two weeks, how often have you been bothered by: 1) feeling nervous, anxious or on edge; 2) not being able to stop or control worrying; 3) feeling down, depressed or hopeless; 4) having little interest or pleasure in doing things?”  Four response options (0-3): Not at all; Several days; More than half the days; Nearly every day | Sum of all four values, categorized as: normal (0-2); mild (3-5); moderate (6-8); severe (9-12). |
| Current incident case count | Number of positive SARS-CoV-2 tests each day as reported to national government. | Natural log of the rolling total of cases reported in past seven days |
| Time | Date of interview | Calendar month of interview, from April 2021 |
