# Supplementary material for: COVID-19 vaccine uptake, confidence and hesitancy in rural KwaZulu-Natal, South Africa between April 2021 and April 2022: A continuous cross-sectional surveillance study
Source: PLOS Glob Public Health. 2023 Jun 27;3(6):e0002033. doi: 10.1371/journal.pgph.0002033 (PMC10298801; doi:10.1371/journal.pgph.0002033)
Supplement: S2 Table — (DOCX) [file pgph.0002033.s003.docx]

**Supplementary Material**

Title: COVID-19 vaccine uptake, confidence and hesitancy in rural KwaZulu-Natal, South Africa between April 2021 and April 2022: a continuous cross-sectional surveillance study

**Authors**: Rachael Piltch-Loeb, Lusanda Mazibuko, Eva Stanton, Thobeka Mngomezulu, Dickman Gareta, Siyabonga Nxumalo, John D. Kraemer, Kobus Herbst, Mark J. Siedner, Guy Harling

**Supplementary Table 2. Comparison of covariate values for retained (n=10,011) and dropped (n=5698) individuals**

|  | N missing | N dropped |  | Dropped | Retained | p-value |
| --- | --- | --- | --- | --- | --- | --- |
| Female | 0 | 5698 |  | 65.8% | 63.6% | <0.001 |
| Age group | 0 | 5698 |  |  |  |  |
| 18-34 |  |  |  | 40.7% | 46.6% | <0.001 |
| 35-49 |  |  |  | 24.0% | 23.1% |  |
| 50-59 |  |  |  | 13.2% | 10.7% |  |
| 60+ |  |  |  | 22.1% | 19.7% |  |
| COVID-19 information sources | 4,634 | 1064 |  |  |  |  |
| Traditional |  |  |  | 98.2% | 97.8% | 0.30 |
| Personal network |  |  |  | 4.8% | 5.1% | 0.61 |
| Healthcare |  |  |  | 15.7% | 15.6% | 0.91 |
| Community |  |  |  | 3.6% | 3.1% | 0.36 |
| Mistrust in government (raw score) | 45 | 5653 |  | 6 (3-7) | 6 (3-7) | 0.018 |
| Highest educational attainment | 1,250 | 4448 |  |  |  |  |
| None |  |  |  | 10.7% | 9.3% | <0.001 |
| Primary |  |  |  | 15.1% | 12.8% |  |
| Some secondary |  |  |  | 43.8% | 46.8% |  |
| Completed secondary |  |  |  | 23.6% | 24.4% |  |
| Any tertiary |  |  |  | 6.7% | 6.7% |  |
| Urbanicity of household | 0 | 5698 |  |  |  |  |
| Peri-Urban |  |  |  | 25.0% | 25.6% | 0.011 |
| Rural |  |  |  | 67.5% | 67.6% |  |
| Urban |  |  |  | 7.6% | 6.8% |  |
| Change in economic stability | 4,636 | 1062 |  |  |  |  |
| Much better off |  |  |  | 0.6% | 0.7% | 0.90 |
| A little better off |  |  |  | 3.6% | 4.1% |  |
| About the same |  |  |  | 79.5% | 79.2% |  |
| A little worse off |  |  |  | 7.8% | 7.6% |  |
| Much worse off |  |  |  | 8.4% | 8.4% |  |
| Change in community wellbeing | 4,636 | 1062 |  |  |  |  |
| Got better |  |  |  | 1.7% | 1.6% | 0.50 |
| Stayed the same |  |  |  | 79.9% | 78.7% |  |
| Got worse |  |  |  | 18.4% | 19.7% |  |
| Has household member aged 60+ | 340 | 5358 |  | 54.5% | 52.9% | 0.004 |
| COVID stereotype stigma score | 4,636 | 1062 |  | 6 (6-6) | 6 (6-6) | 0.94 |
| COVID anticipated stigma score | 4,636 | 1062 |  | 5 (3-5) | 5 (3-5) | 0.97 |
| Future COVID infection concern level | 32 | 5666 |  |  |  |  |
| Not at all |  |  |  | 21.4% | 20.7% | 0.001 |
| Slightly concerned |  |  |  | 39.1% | 39.7% |  |
| Moderately concerned |  |  |  | 17.5% | 18.7% |  |
| Very concerned |  |  |  | 22.0% | 20.9% |  |
| Knows someone who has had COVID | 51 | 5647 |  | 5.8% | 5.0% | 0.002 |
| Any other household members vaccinated | 0 | 5698 |  | 30.6% | 35.8% | <0.001 |
| PHQ-4 categories | 99 | 5599 |  |  |  |  |
| Normal |  |  |  | 65.2% | 63.2% | <0.001 |
| Mild |  |  |  | 19.1% | 17.9% |  |
| Moderate |  |  |  | 12.8% | 15.1% |  |
| Severe |  |  |  | 3.0% | 3.7% |  |
| Past 7-day KZN case count per 1000 population | 0 | 5698 |  | 0.17 (0.08-0.23) | 0.15 (0.06-0.22) | <0.001 |
| COVID vaccine willingness | 0 | 5698 |  |  |  |  |
| Already vaccinated |  |  |  | 4.1% | 5.2% |  |
| Definitely would |  |  |  | 24.5% | 22.7% |  |
| Probably would |  |  |  | 32.6% | 32.4% |  |
| Probably would not |  |  |  | 26.3% | 29.6% |  |
| Definitely would not |  |  |  | 7.9% | 6.6% |  |
