# Supplementary material for: COVID-19 vaccine uptake, confidence and hesitancy in rural KwaZulu-Natal, South Africa between April 2021 and April 2022: A continuous cross-sectional surveillance study
Source: PLOS Glob Public Health. 2023 Jun 27;3(6):e0002033. doi: 10.1371/journal.pgph.0002033 (PMC10298801; doi:10.1371/journal.pgph.0002033)
Supplement: S3 Table — (DOCX) [file pgph.0002033.s004.docx]

**Supplementary Material**

Title: COVID-19 vaccine uptake, confidence and hesitancy in rural KwaZulu-Natal, South Africa between April 2021 and April 2022: a continuous cross-sectional surveillance study

**Authors**: Rachael Piltch-Loeb, Lusanda Mazibuko, Eva Stanton, Thobeka Mngomezulu, Dickman Gareta, Siyabonga Nxumalo, John D. Kraemer, Kobus Herbst, Mark J. Siedner, Guy Harling

**Supplementary Table 3. Descriptive statistics for the sample by month of interview**

|  | **Total** |  | **Apr-21** | **May-21** | **Jun-21** | **Jul-21** | **Aug-21** | **Sep-21** | **Oct-21** | **Nov-21** | **Dec-21** | **Jan-22** | **Feb-22** | **Mar-22** | **Apr-22** |
| --- | --- | --- | --- | --- | --- | --- | --- | --- | --- | --- | --- | --- | --- | --- | --- |
| N | 10,011 |  | 367 | 1,196 | 581 |  | 454 | 679 | 1,194 | 1,369 | 383 | 626 | 1,146 | 1,664 | 352 |
| Female | 64.8% |  | 75.2% | 74.2% | 62.1% |  | 59.9% | 60.4% | 65.9% | 68.0% | 70.8% | 65.5% | 67.8% | 66.7% | 64.8% |
| Age group |  |  |  |  |  |  |  |  |  |  |  |  |  |  |  |
| 18-34 | 41.8% |  | 37.9% | 32.6% | 42.5% |  | 45.8% | 42.6% | 36.2% | 35.4% | 37.9% | 39.1% | 31.1% | 39.5% | 41.8% |
| 35-49 | 20.7% |  | 22.1% | 21.4% | 22.7% |  | 33.3% | 25.0% | 25.9% | 23.2% | 30.0% | 25.6% | 25.3% | 24.0% | 20.7% |
| 50-59 | 13.1% |  | 15.0% | 14.4% | 12.7% |  | 11.5% | 15.6% | 15.9% | 15.7% | 14.6% | 15.2% | 16.2% | 13.5% | 13.1% |
| 60+ | 24.4% |  | 25.1% | 31.6% | 22.0% |  | 9.5% | 16.8% | 22.0% | 25.8% | 17.5% | 20.1% | 27.4% | 23.0% | 24.4% |
| COVID-19 information sources |  |  |  |  |  |  |  |  |  |  |  |  |  |  |  |
| Traditional | 97.4% |  | 98.1% | 99.1% | 98.1% |  | 98.9% | 99.6% | 98.1% | 98.2% | 98.2% | 97.9% | 98.3% | 97.7% | 97.4% |
| Personal network | 4.0% |  | 10.1% | 2.9% | 4.3% |  | 4.2% | 4.7% | 3.4% | 3.6% | 7.3% | 4.8% | 7.5% | 4.7% | 4.0% |
| Healthcare | 21.6% |  | 14.4% | 11.8% | 6.2% |  | 13.4% | 12.8% | 19.3% | 16.2% | 16.7% | 22.0% | 17.5% | 16.0% | 21.6% |
| Community | 2.6% |  | 6.0% | 6.7% | 4.3% |  | 4.8% | 2.1% | 3.6% | 2.6% | 2.9% | 3.7% | 2.5% | 3.1% | 2.6% |
| Mistrust in government (raw score) | 6 (5-8) |  | 7 (6-9) | 7 (5-9) | 6 (3-9) |  | 6 (3-8) | 6 (5-9) | 6 (3-7) | 6 (3-6) | 6 (3-6) | 6 (3-6) | 6 (3-6) | 6 (3-6) | 6 (5-8) |
| Highest educational attainment |  |  |  |  |  |  |  |  |  |  |  |  |  |  |  |
| None | 15.9% |  | 11.4% | 17.1% | 11.7% |  | 7.3% | 4.6% | 9.1% | 13.9% | 8.9% | 5.3% | 12.4% | 11.1% | 15.9% |
| Primary | 18.2% |  | 20.4% | 19.2% | 16.0% |  | 9.3% | 10.0% | 15.8% | 17.6% | 14.4% | 11.7% | 18.7% | 16.7% | 18.2% |
| Some secondary | 44.3% |  | 39.5% | 41.0% | 43.7% |  | 50.0% | 44.0% | 39.4% | 44.3% | 46.0% | 39.5% | 41.8% | 42.5% | 44.3% |
| Completed secondary | 15.9% |  | 22.9% | 19.8% | 23.9% |  | 26.7% | 26.2% | 27.2% | 19.3% | 22.2% | 32.9% | 21.9% | 23.1% | 15.9% |
| Any tertiary | 5.7% |  | 5.7% | 2.9% | 4.6% |  | 6.8% | 15.2% | 8.5% | 5.0% | 8.6% | 10.7% | 5.2% | 6.7% | 5.7% |
| Urbanicity of household |  |  |  |  |  |  |  |  |  |  |  |  |  |  |  |
| Peri-Urban | 1.4% |  | 25.6% | 0.5% | 7.2% |  | 26.2% | 56.4% | 55.2% | 10.2% | 48.0% | 55.6% | 26.3% | 10.9% | 1.4% |
| Rural | 98.6% |  | 74.4% | 99.5% | 92.8% |  | 34.4% | 9.6% | 38.0% | 88.2% | 21.1% | 22.4% | 71.3% | 88.6% | 98.6% |
| Urban | 0.0% |  | 0.0% | 0.0% | 0.0% |  | 39.4% | 34.0% | 6.8% | 1.7% | 30.8% | 22.0% | 2.4% | 0.4% | 0.0% |
| Change in economic stability |  |  |  |  |  |  |  |  |  |  |  |  |  |  |  |
| Much better off | 0.0% |  | 0.8% | 0.9% | 0.0% |  | 0.7% | 0.3% | 0.3% | 0.9% | 0.3% | 0.8% | 0.4% | 0.6% | 0.0% |
| A little better off | 2.3% |  | 7.9% | 4.4% | 3.3% |  | 5.1% | 2.9% | 4.4% | 2.9% | 3.1% | 4.6% | 2.0% | 3.1% | 2.3% |
| About the same | 83.2% |  | 71.7% | 78.1% | 76.9% |  | 75.6% | 82.0% | 79.0% | 80.0% | 76.2% | 80.2% | 81.5% | 82.0% | 83.2% |
| A little worse off | 8.2% |  | 15.5% | 12.0% | 11.5% |  | 9.7% | 7.4% | 5.5% | 6.5% | 6.5% | 7.5% | 5.8% | 6.2% | 8.2% |
| Much worse off | 6.3% |  | 4.1% | 4.5% | 8.3% |  | 9.0% | 7.4% | 10.8% | 9.6% | 13.8% | 6.9% | 10.3% | 8.2% | 6.3% |
| Change in community wellbeing |  |  |  |  |  |  |  |  |  |  |  |  |  |  |  |
| Got better | 2.0% |  | 4.4% | 1.4% | 0.9% |  | 2.0% | 1.9% | 1.5% | 2.0% | 2.6% | 1.6% | 1.5% | 1.4% | 2.0% |
| Stayed the same | 81.0% |  | 67.6% | 80.4% | 80.4% |  | 76.9% | 80.3% | 84.6% | 79.8% | 72.1% | 82.3% | 76.9% | 83.1% | 81.0% |
| Got worse | 17.0% |  | 28.1% | 18.2% | 18.8% |  | 21.1% | 17.8% | 13.9% | 18.2% | 25.3% | 16.1% | 21.6% | 15.5% | 17.0% |
| Has household member aged 60+ | 58.5% |  | 61.3% | 67.7% | 61.4% |  | 34.8% | 43.9% | 52.0% | 58.2% | 40.7% | 46.3% | 57.1% | 58.5% | 58.5% |
| COVID stereotype stigma score | 6 (6-6) |  | 6 (6-8) | 6 (6-7) | 6 (6-7) |  | 6 (6-6) | 6 (6-6) | 6 (5-6) | 6 (6-6) | 6 (6-6) | 6 (6-6) | 6 (6-6) | 6 (6-6) | 6 (6-6) |
| COVID anticipated stigma score | 5 (3-5) |  | 5 (5-7) | 5 (3-5) | 5 (3-5) |  | 5 (2-5) | 5 (1-5) | 5 (1-5) | 5 (4-5) | 5 (4-5) | 5 (3-5) | 5 (4-5) | 5 (3-5) | 5 (3-5) |
| Future COVID infection concern level |  |  |  |  |  |  |  |  |  |  |  |  |  |  |  |
| Not at all | 16.8% |  | 24.0% | 24.4% | 18.9% |  | 13.0% | 22.4% | 21.8% | 23.0% | 26.1% | 25.9% | 22.4% | 19.8% | 16.8% |
| Slightly concerned | 56.0% |  | 27.8% | 26.8% | 24.4% |  | 37.2% | 34.9% | 37.9% | 41.6% | 33.9% | 38.8% | 44.3% | 48.7% | 56.0% |
| Moderately concerned | 17.0% |  | 12.8% | 17.0% | 13.8% |  | 18.7% | 17.5% | 17.3% | 15.8% | 13.3% | 15.0% | 17.4% | 19.2% | 17.0% |
| Very concerned | 10.2% |  | 35.4% | 31.8% | 42.9% |  | 31.1% | 25.2% | 23.1% | 19.6% | 26.6% | 20.3% | 15.9% | 12.4% | 10.2% |
| Knows someone who has had COVID | 5.1% |  | 13.9% | 7.4% | 5.9% |  | 9.7% | 10.2% | 6.8% | 4.8% | 3.9% | 6.1% | 4.2% | 4.6% | 5.1% |
| Any other household members vaccinated | 47.2% |  | 0.5% | 0.4% | 8.4% |  | 9.7% | 26.7% | 25.7% | 38.8% | 29.5% | 32.1% | 37.1% | 44.3% | 47.2% |
|  |  |  |  |  |  |  |  |  |  |  |  |  |  |  |  |
|  |  |  |  |  |  |  |  |  |  |  |  |  |  |  |  |
|  |  |  |  |  |  |  |  |  |  |  |  |  |  |  |  |
| PHQ-4 categories |  |  |  |  |  |  |  |  |  |  |  |  |  |  |  |
| Normal | 76.4% |  | 51.0% | 50.9% | 60.9% |  | 56.4% | 57.0% | 68.4% | 73.3% | 76.8% | 74.0% | 69.5% | 71.8% | 76.4% |
| Mild | 13.1% |  | 30.2% | 37.6% | 25.5% |  | 33.3% | 25.2% | 14.8% | 12.9% | 8.4% | 12.1% | 15.1% | 15.6% | 13.1% |
| Moderate | 9.7% |  | 9.5% | 8.1% | 10.8% |  | 7.5% | 16.1% | 14.2% | 11.0% | 12.8% | 12.0% | 12.7% | 11.0% | 9.7% |
| Severe | 0.9% |  | 9.3% | 3.3% | 2.8% |  | 2.9% | 1.8% | 2.5% | 2.8% | 2.1% | 1.9% | 2.7% | 1.7% | 0.9% |
| COVID vaccine willingness |  |  |  |  |  |  |  |  |  |  |  |  |  |  |  |
| Already vaccinated | 26.7% |  | 0.0% | 0.9% | 9.1% |  | 12.8% | 31.8% | 29.1% | 36.5% | 32.1% | 34.7% | 36.0% | 31.8% | 26.7% |
| Definitely would | 11.9% |  | 31.6% | 47.7% | 52.8% |  | 55.9% | 35.2% | 37.5% | 28.9% | 33.2% | 26.4% | 26.3% | 18.1% | 11.9% |
| Probably would | 42.9% |  | 21.8% | 21.3% | 16.7% |  | 17.2% | 24.0% | 20.4% | 21.6% | 24.5% | 24.1% | 22.0% | 34.9% | 42.9% |
| Probably would not | 9.7% |  | 20.2% | 15.0% | 9.5% |  | 7.7% | 5.2% | 6.0% | 5.8% | 6.3% | 8.3% | 7.9% | 8.5% | 9.7% |
| Definitely would not | 8.8% |  | 26.4% | 15.1% | 11.9% |  | 6.4% | 3.8% | 6.9% | 7.2% | 3.9% | 6.5% | 7.8% | 6.6% | 8.8% |

Notes: No in-person interviews were conducted in July in response to concern regarding rising case numbers locally. Tests of homogeneity across all months were rejected for all variables with p-value <0.001, with the exception traditional COVID-19 information sources (p=0.086).
