# Supplementary material for: COVID-19 vaccine uptake, confidence and hesitancy in rural KwaZulu-Natal, South Africa between April 2021 and April 2022: A continuous cross-sectional surveillance study
Source: PLOS Glob Public Health. 2023 Jun 27;3(6):e0002033. doi: 10.1371/journal.pgph.0002033 (PMC10298801; doi:10.1371/journal.pgph.0002033)
Supplement: S4 Table — (DOCX) [file pgph.0002033.s005.docx]

**Supplementary Material**

Title: COVID-19 vaccine uptake, confidence and hesitancy in rural KwaZulu-Natal, South Africa between April 2021 and April 2022: a continuous cross-sectional surveillance study

**Authors**: Rachael Piltch-Loeb, Lusanda Mazibuko, Eva Stanton, Thobeka Mngomezulu, Dickman Gareta, Siyabonga Nxumalo, John D. Kraemer, Kobus Herbst, Mark J. Siedner, Guy Harling

**Supplementary Table 4. Sensitivity analysis multivariable regressions**

| **Dependent variable** | **Primary  analysis** | |  | **Any  negative** | |  | **Definitely negative** | |  |
| --- | --- | --- | --- | --- | --- | --- | --- | --- | --- |
| Interview Month (vs April 2021) | 1.00 |  |  | 1.00 |  |  | 1.00 |  | |
| May 2021 | 0.59 | [0.45, 0.76] |  | 0.53 | [0.40, 0.70] |  | 0.50 | [0.34, 0.73] | |
| June 2021 | 0.35 | [0.26, 0.46] |  | 0.37 | [0.26, 0.51] |  | 0.47 | [0.30, 0.75] | |
| August 2021 | 0.27 | [0.14, 0.51] |  | 0.32 | [0.11, 0.94] |  | 0.58 | [0.11, 2.94] | |
| September 2021 | 0.17 | [0.12, 0.23] |  | 0.13 | [0.08, 0.21] |  | 0.14 | [0.07, 0.29] | |
| October 2021 | 0.21 | [0.16, 0.28] |  | 0.20 | [0.14, 0.28] |  | 0.23 | [0.14, 0.37] | |
| November 2021 | 0.22 | [0.16, 0.28] |  | 0.24 | [0.17, 0.33] |  | 0.29 | [0.19, 0.45] | |
| December 2021 | 0.21 | [0.15, 0.31] |  | 0.19 | [0.11, 0.32] |  | 0.18 | [0.08, 0.40] | |
| January 2022 | 0.24 | [0.17, 0.34] |  | 0.28 | [0.18, 0.43] |  | 0.29 | [0.15, 0.53] | |
| February 2022 | 0.22 | [0.17, 0.29] |  | 0.29 | [0.21, 0.40] |  | 0.31 | [0.19, 0.52] | |
| March 2022 | 0.29 | [0.22, 0.38] |  | 0.27 | [0.19, 0.36] |  | 0.24 | [0.15, 0.39] | |
| April 2022 | 0.42 | [0.30, 0.58] |  | 0.33 | [0.22, 0.49] |  | 0.33 | [0.18, 0.60] | |
| Female vs Male | 0.70 | [0.65, 0.76] |  | 0.78 | [0.70, 0.88] |  | 0.78 | [0.67, 0.91] | |
| Age (vs 18-34 year olds) | 1.00 |  |  | 1.00 |  |  | 1.00 |  | |
| 35-49 years | 0.65 | [0.59, 0.72] |  | 0.84 | [0.73, 0.97] |  | 0.75 | [0.62, 0.91] | |
| 50-59 years | 0.42 | [0.37, 0.47] |  | 0.62 | [0.51, 0.75] |  | 0.58 | [0.45, 0.76] | |
| 60 years and older | 0.21 | [0.19, 0.25] |  | 0.55 | [0.45, 0.68] |  | 0.52 | [0.39, 0.70] | |
| COVID information sources | 1.00 |  |  | 1.00 |  |  | 1.00 |  | |
| Traditional | 0.88 | [0.66, 1.18] |  | 1.02 | [0.66, 1.57] |  | 0.85 | [0.49, 1.46] | |
| Personal network | 1.06 | [0.89, 1.26] |  | 1.03 | [0.80, 1.33] |  | 1.04 | [0.75, 1.44] | |
| Healthcare | 1.09 | [0.98, 1.20] |  | 1.10 | [0.94, 1.28] |  | 1.10 | [0.90, 1.34] | |
| Community | 1.15 | [0.95, 1.40] |  | 1.13 | [0.86, 1.49] |  | 1.10 | [0.77, 1.56] | |
| Mistrust in government, z-score | 1.47 | [1.42, 1.53] |  | 1.54 | [1.46, 1.63] |  | 1.52 | [1.41, 1.63] | |
| Highest educational attainment (vs None) | 1.00 |  |  | 1.00 |  |  | 1.00 |  | |
| Primary | 0.91 | [0.78, 1.05] |  | 0.92 | [0.74, 1.16] |  | 1.00 | [0.74, 1.35] | |
| Some secondary | 0.78 | [0.67, 0.91] |  | 0.89 | [0.71, 1.12] |  | 0.83 | [0.60, 1.13] | |
| Completed secondary | 0.69 | [0.59, 0.82] |  | 0.81 | [0.63, 1.05] |  | 0.86 | [0.61, 1.21] | |
| Any tertiary | 0.63 | [0.51, 0.77] |  | 0.73 | [0.53, 1.00] |  | 0.79 | [0.52, 1.22] | |
| Household location (vs rural) | 1.00 |  |  | 1.00 |  |  | 1.00 |  | |
| Peri-Urban | 1.12 | [0.98, 1.27] |  | 1.17 | [0.98, 1.40] |  | 1.31 | [1.03, 1.69] | |
| Urban | 0.96 | [0.74, 1.25] |  | 1.02 | [0.74, 1.40] |  | 0.90 | [0.53, 1.52] | |
| Household economic change (vs None) | 1.00 |  |  | 1.00 |  |  | 1.00 |  | |
| Much better off | 1.13 | [0.69, 1.83] |  | 1.52 | [0.82, 2.80] |  | 0.53 | [0.18, 1.50] | |
| A little better off | 1.08 | [0.89, 1.31] |  | 0.65 | [0.47, 0.89] |  | 0.94 | [0.65, 1.38] | |
| A little worse off | 1.00 | [0.87, 1.15] |  | 0.94 | [0.76, 1.16] |  | 1.01 | [0.77, 1.34] | |
| Much worse off | 0.90 | [0.77, 1.05] |  | 0.65 | [0.50, 0.84] |  | 0.68 | [0.48, 0.98] | |
| Community wellbeing change (vs None) | 1.00 |  |  | 1.00 |  |  | 1.00 |  | |
| Got better | 0.99 | [0.75, 1.31] |  | 0.86 | [0.55, 1.35] |  | 1.16 | [0.68, 1.98] | |
| Got worse | 1.06 | [0.95, 1.19] |  | 0.97 | [0.82, 1.15] |  | 0.77 | [0.61, 0.97] | |
| Household member aged 60+ | 1.04 | [0.95, 1.13] |  | 0.99 | [0.87, 1.13] |  | 0.90 | [0.76, 1.07] | |
| COVID stereotype stigma, z-score | 1.01 | [0.96, 1.06] |  | 1.04 | [0.97, 1.13] |  | 1.00 | [0.91, 1.11] | |
| COVID anticipated stigma, z-score | 1.02 | [0.97, 1.06] |  | 1.04 | [0.98, 1.11] |  | 1.03 | [0.95, 1.12] | |
| KZN cases per 1000 pop in past 7 days | 0.80 | [0.53, 1.21] |  | 0.33 | [0.22, 0.49] |  | 0.33 | [0.18, 0.60] | |
| Concern if got COVID (vs Not at all) | 1.00 |  |  | 1.00 |  |  | 1.00 |  | |
| Slightly concerned | 1.35 | [1.22, 1.50] |  | 0.94 | [0.81, 1.10] |  | 0.76 | [0.62, 0.92] | |
| Moderately concerned | 1.38 | [1.21, 1.56] |  | 1.27 | [1.07, 1.52] |  | 0.83 | [0.66, 1.05] | |
| Very concerned | 0.84 | [0.75, 0.94] |  | 0.96 | [0.81, 1.14] |  | 0.54 | [0.43, 0.67] | |
| Knows someone who has had COVID | 0.69 | [0.59, 0.80] |  | 0.73 | [0.58, 0.93] |  | 0.77 | [0.56, 1.05] | |
| Any other household members vaccinated | 0.65 | [0.59, 0.71] |  | 0.71 | [0.61, 0.82] |  | 0.66 | [0.54, 0.82] | |
| PHQ-4 (vs normal) | 1.00 |  |  | 1.00 |  |  | 1.00 |  | |
| Mild | 0.96 | [0.87, 1.06] |  | 0.73 | [0.63, 0.85] |  | 0.94 | [0.77, 1.15] | |
| Moderate | 0.91 | [0.80, 1.03] |  | 0.94 | [0.77, 1.14] |  | 0.85 | [0.65, 1.13] | |
| Severe | 1.16 | [0.91, 1.47] |  | 1.34 | [0.98, 1.83] |  | 2.22 | [1.55, 3.18] | |
| Village level variance | 1.02 |  |  | 1.01 |  |  | 1.05 |  | |
