# Supplementary material for: COVID-19 vaccine uptake, confidence and hesitancy in rural KwaZulu-Natal, South Africa between April 2021 and April 2022: A continuous cross-sectional surveillance study
Source: PLOS Glob Public Health. 2023 Jun 27;3(6):e0002033. doi: 10.1371/journal.pgph.0002033 (PMC10298801; doi:10.1371/journal.pgph.0002033)
Supplement: Attachment — Submitted filename: AHRI Covid Vaccine 2021-22 - R1 response - 15May2023.docx [file pgph.0002033.s006.docx]

**PGPH-D-23-00155**

**COVID-19 vaccine uptake, confidence and hesitancy in rural KwaZulu-Natal, South Africa between April 2021 and April 2022: a continuous cross-sectional surveillance study**

PLOS Global Public Health - PGPH-D-23-00155. Review dated 10 April 2023

We thank the reviewers and editor for their comments and the opportunity to revise and strengthen this manuscript. We have responded to all comments, as outlined below.

**Editor/Reviewer comments** are in bold text, our response is in normal text with text additions underlined and ~~removed text~~ shown with strikethrough when relevant. All page numbers for paper edits refer to the revised manuscript version without tracked changes.

Journal Requirements

**Please include the following request in the decision letter, and ping me (hlandenmark@plos.org) with follow up. “Please include a complete copy of PLOS’ questionnaire on inclusivity in global research in your revised manuscript. Our policy for research in this area aims to improve transparency in the reporting of research performed outside of researchers’ own country or community. The policy applies to researchers who have travelled to a different country to conduct research, research with Indigenous populations or their lands, and research on cultural artefacts. The questionnaire can also be requested at the journal’s discretion for any other submissions, even if these conditions are not met. Please find more information on the policy and a link to download a blank copy of the questionnaire here: https://journals.plos.org/plosone/s/best-practices-in-research-reporting. Please upload a completed version of your questionnaire as Supporting Information when you resubmit your manuscript.**

We have reviewed this questionnaire and included a copy as part of our resubmission. We have added a note to this in the main text at the end of the Methods section:

“Inclusivity in global research

Additional information regarding the ethical, cultural, and scientific considerations specific to inclusivity in global research is included as Supplementary Table 2.”

**Please review your reference list to ensure that it is complete and correct. If you have cited papers that have been retracted, please include the rationale for doing so in the manuscript text, or remove these references and replace them with relevant current references. Any changes to the reference list should be mentioned in the rebuttal letter that accompanies your revised manuscript. If you need to cite a retracted article, indicate the article’s retracted status in the References list and also include a citation and full reference for the retraction notice.**

We have reviewed our reference list; there are no retracted articles included. Any changes are noted in this document.

Reviewer #1

**The authors have conducted a semi- longitudinal study to assess the COVID 19 vaccine uptake, confidence and hesitancy in rural Kwazulu-Natal, South Africa. From an initial sample of 15755 persons surveyed a sub-sample of 10 011 was retained for analyses after eliminating subjects who did not fully comply with the inclusion criteria. The WHO recommended determinants/drivers of Vaccine hesitancy were interrogated, and these included, age gender, level of education, socioeconomic status etc. The authors stated that they had performed regression analysis on their data without specifying the software employed, which would make verification difficult.**

We apologise for this omission, which has now been addressed in the revised manuscript:

“All statistical analyses were conducted in Stata 15.1.”

**The results obtained were generally in agreement with those obtained in South Africa and other African countries, although vaccine acceptance was relatively higher in South Africa than in most of Sub-Saharan Africa. The main drivers of vaccine hesitancy were mistrust of the Government, followed by low level of education and being male. Curiously enough the authors did not explore the role of social media anti-vaccine mis/disinformation which was reported as a major vaccine hesitancy driver in many studies including some of those cited by the authors of this study.**

We appreciate the reviewer’s comment and agree that misinformation on social media has been associated with vaccine hesitancy in many studies. In our questionnaire, we did not distinguish where vaccine claims may have come from. Our questions related to social media were limited to trust in social media. Mis/disinformation encountered on social media may have contributed to mistrust in government, among other vaccine perceptions. Because this study was not initiated to ask about specific vaccine-related narratives, we are not able to distinguish where stories about vaccines came from and how vaccine perceptions arose. We therefore, focused on the available data and examined the role of the effects of information exposure such as perceptions of government and the vaccine. We have added a line in our limitations section to note this limitation:

“We recognize there may be other factors that impact vaccine hesitancy which we did not include in our questionnaire. For example, our study did not include survey items related to social media usage or specific pieces of mis/disinformation the user may have encountered.”

**I am also concerned that the results are presented mainly as three crowded tables full of raw data making reading rather difficult. The authors should consider using histograms and pie diagrams in addition to the tables to illustrate some of their findings.**

We agree with the reviewer that the results tables include extensive data, which can be challenging to immediately interpret. We have therefore summarized our central findings as the reviewer suggests also using figures. For example, in Figure 2 we demonstrate how our primary outcome, i.e., vaccine willingness levels differs across time and in Figure 3 we demonstrate how vaccine hesitancy varies across key explanatory variables.

**In conclusion, I am satisfied that this study that was focused on a rural sample will complement the findings of other studies in urban areas. I therefore recommend that the paper be accepted subject to satisfactorily addressing the issues that I have raised above.**

Reviewer #2

**This article describes the findings from a cross-sectional surveillance study evaluating COVID-19 vaccine update, confidence, and hesitancy in a defined region in KwaZulu-Natal, South Africa. The authors were able to utilize an existing survey infrastructure to gather additional data about COVID-19 vaccine hesitancy and accumulate a large sample size of over 10,000 responses. They identified key contributors to vaccine hesitancy in this region, including age of individuals and proximity to others with experience with COVID-19 vaccination and/or COVID-19 disease.**

**Overall the manuscript is quite strong, with sample size providing sufficient statistical power to interrogate individual parameters as well as overall trends. The utilization of the WHO framework for addressing vaccine hesitancy enables the results to be compared to other analyses. The use of a pre-existing (prior to COVID) survey cohort is especially interesting as it lends validity to the interpretation of results in terms of the independence of the participants from actual vaccination clinics or healthcare settings, as well as the (presumed) established trust between those responding to the surveys and those administering them. The inclusion of a wide range of ages and level of education also lend broader interpretability of the data, though the restriction to this one location is acknowledged by both the authors and this reviewer. The authors do a good job of placing their findings in context and relating to the current literature on vaccine hesitancy.**

We thank the reviewer for their positive comments.

**A few of the survey questions were structured in a way that could be considered leading. As an examples, the three Mistrust in government questions were phrased in a decidedly anti-government way. However, given the large literature on the mistrust of government of individuals in the population that contributed responses for this analysis I do not believe this directly influenced the results in a meaningful way. I could not easily locate the source of these questions in the sources for the instruments referenced to see if this had previously been addressed.**

We appreciate the reviewer’s insight here, and agree that the questions are framed in an anti-government way. Unfortunately these concepts are challenging to ask about and inherently are asked with a pro-government or anti-government bend. For example, in prior work by the research team we have also asked similar survey items in different ways. In a study Dr. Piltch-Loeb was a part of (Savoia et al, 2021 <https://doi.org/10.3390/vaccines10050671>), the team asked: “Do you feel you are receiving fully transparent information about the COVID-19 situation from your national government officials?” Often respondents are asked for their level of agreement with statements that suggest positive feelings about the government response to Covid-19 (e.g., the 2020 seminal study by Lazarus et al <https://doi.org/10.1371/journal.pone.0240011>). With either framing, it is incumbent upon the respondent to interpret the survey item and respond with an ordinal level of agreement. Given the large literature that has emerged on mistrust in government, despite many studies using this positive bend related to COVID-19, we agree with the reviewer it is unlikely that the framing of these items unduly influenced trust in government.

The items in this survey were adopted by this study as part of a multi-site effort to generate comparable data across South Africa (the SAPRIN network, <https://doi.org/10.1093/ije/dyab261>). To our knowledge they were not derived from past instruments, but instead developed rapidly in 2020 as the Covid-19 pandemic emerged. They have been subsequently retained to maintain consistency over place and time.

Nonetheless, to highlight this as a potential source of bias, we have added text to the limitations section about this consideration:

“We note that some of the survey items, e.g., those relating to mistrust in government and Covid-related stigma, were not fully validated given the speed with which they were developed in the pandemic; further work is needed to validate and replicate the associations seen for these parameters.”

**(2) Given the finding that distrust in the government was associated with hesitancy, and that government websites were included among the Traditional Sources of information, which was the category that received an overwhelmingly high response to the question "Where do you get information about COVID-19 that you trust?", the analysis may benefit from separating out government websites to a different category, or at least providing percentages of response for the different subtypes (newspapers, radio, TV, government websites) to see the contribution the government websites made to that category.**

We double-checked the data and found that only 112 of the 10,011 respondents (or 1.1%) reported using government websites as a source for Covid-19 information that they trust. Slightly more used newspapers or news websites (7.3%), with the overwhelming majority reporting getting information either from TV (81.2%) or radio (87.2%). As a result, government website use adds only 7 individuals to the positive responses for this category, making the potential for affecting our results minimal. We now outline these matters in the Results to make the composition of the variable clearer:

“Almost everyone relied on traditional information sources for COVID-19 content (98.3%, of which the great majority used radio or television, with only 7.3% using newspapers and 1.1% government websites), with a minority (15.7%) also using healthcare sources.”

**(3) There are a few missing words in the abstract and introduction sections, though I didn't see any or as many in the remaining section.**

We have made several minor text edits to the abstract and introduction. Thank you.

**Overall this is a very interesting study with informative results that will help inform the rollout of future vaccination campaigns, either for COVID-19 or other vaccines, and possibly other health interventions.**
